# Gel Evolution of Copper Tailing-Based Green Geopolymers in Marine Related Environments

**DOI:** 10.3390/ma15134599

**Published:** 2022-06-30

**Authors:** Jing Li, Lang Yang, Feng Rao, Xiang Tian

**Affiliations:** 1 Zijin School of Geology and Mining, Fuzhou University, Fuzhou 350108, China; lijing610082858@163.com (J.L.); siryanglang@fzu.edu.cn (L.Y.); 2 School of Civil Engineering, Changsha University of Science and Technology, Changsha 410114, China

**Keywords:** geopolymer, marine concrete, compressive strength, carbonization, corrosion resistance

## Abstract

Geopolymers have attracted extensive attention in the marine environment because of its special reticulate nanostructure. Gel evolutions of copper tailing-based green geopolymers were studied under air, deionized water, seawater, freeze–thaw cycle and carbonization environments. Their mechanical properties and microstructures were characterized by compressive strength measurement, X-ray diffraction (XRD), Fourier-transform infrared spectroscopy (FTIR), nuclear magnetic resonance (NMR) and scanning electron microscopy (SEM). It was found that the deionized water and natural marine water exposure promoted the evolution degree of geopolymers and improved their compressive strength, while exposure to the carbonization environment weakened the gel evolution and decreased the cross-linking degree of the Sodium aluminosilicate hydrate (N-A-S-H)gel structure, resulting in a decline of compressive strength. The geopolymer exposed in the freeze–thaw cycle exhibited the worst deterioration due to the expansion caused by the crystallization in the geopolymer. These results are essential and beneficial to further understanding the gel formation process in various marine environments and could promote the investigation of green concrete.

## 1. Introduction

Geopolymer is an amorphous material of three-dimensional (3D) microstructure in the composition of M_2_O∙mAl_2_O_3_∙nSiO_2_, where M represents alkali metals, m ≈ 1 and 2 ≤ n ≤ 6 [1]. The reactions in the geopolymeric process include decomposition, recombination, polymerization, condensation and solidification. Although Davidovits defended that geopolymers differ from alkali-activated materials (AAMs) in chemistry, molecular structure, long-term durability, etc., plenty of works use the term geopolymer when dealing with AAMs, even in high-calcium alkali-activated systems [2,3]. Geopolymers are mainly studied in the application of green concrete materials for the replacement of ordinary Portland cement (OPC) material [4,5,6], although there are other particular applications of geopolymers that are also interesting, for example, the synthesis of waste-based materials into marine concrete geopolymers [7,8,9].

Compared with normal concrete, marine concrete endures damage from physical forces (e.g., wind load and the impact of waves and currents) and chemical corrosions, which are mainly from chloride and carbide penetration and the corrosion of steel bars, as well as the crystallization of salts in the marine environment. These effects cause cracking and deterioration of the steel bars under freeze–thaw circles [10]. Therefore, the geopolymer has attracted extensive attention due to its superior mechanical characteristics and higher resistance in the marine environment than OPC concrete. The 3D microstructure and compacted tetrahedral-silica and aluminum-structure inner geopolymers enables them to resist exotic physical force and chemical corrosion. Wu et al. [9] successfully synthesized geopolymers under the marine environment with metakaolin and copper slag reprocessing tailings; meanwhile, they found the chemical characteristics of geopolymers were much improved due to the changing Si/Al ratio in the micro-structure, promoting the geopolymerization process. Besides, Tennakoon et al. [11] pointed out that the Cl^−^ diffusion coefficient in geopolymers was much lower than in OPC concrete even under an extended test period. The reinforced bar embedded in geopolymers also displayed higher corrosion resistance than the OPC concrete. After investigating the impact of ions on the performances of geopolymers, Xie et al. [12,13,14] confirmed that the Cl^−^, Mg^2+^ and SO_4_^2−^ had little influence on the rheological and mechanical properties of geopolymers as compared with OPC. Moreover, previous studies reported that the fly ash and slag among the aluminosilicate minerals and industrial wastes could effectively improve the compressive and freezing resistance of the geopolymer, and the slag performed better than the fly ash. This was attributed to the large amount of Ca in slag, which would generate a new hydration product, calcium silicate hydrate (C-A-S-H)gel, and fill in the geopolymer structure, giving the geopolymer a higher density and contributing to its strength development [15]. Thus, slag has attracted more and more attention in the area of geopolymer preparation and application. Recently, the fast development and utilization of mineral resources discharged amounts of tailings and slag, and China’s tailing and slag stockpile reached more than 19.5 billion tons according to China’s Mineral Resources Conservation and Comprehensive Utilization Report (2018) [16]. The massive amount of tailings and slag may cause environmental problems, including the contamination of surrounding air, water bodies and soil [17,18]. Therefore, it is urgent and necessary to reprocess the tailings and slag to reduce their adverse effects. Tailings and slag usually contain a large amount of aluminosilicate and Ca, making them high potential geopolymer raw materials to prepare marine concrete [8]. However, the previous studies always explored one certain special aspect of the tailings-based geopolymers [19,20,21]. Few works focused on their geopolymerization processes and microstructure differences under various exposure conditions [22], especially the natural marine environment. Thus, it is much meaningful and essential to study the gel evolution and microstructure variation of the geopolymer under different natural environments, e.g., in the marine environment, to prepare advanced marine concrete.

In this work, copper tailings (CT) and blast furnace slag (BFS) were used to synthesize the geopolymer. Then, an attempt was made to perform the following: (1) study the geopolymerization process and feasibility of using a copper-tailings-based geopolymer as marine concrete, (2) explore the evolution of the mechanical properties and microstructure of a mine-tailings-based geopolymer concrete in different marine related environments (and better study air and deionized water environments as reference phases), and (3) prepare green marine geopolymer concrete using two solid wastes, CT and BFS, through geopolymeric reaction technology(treating waste with waste provides a reference for realizing the consumption of bulk solid waste).

## 2. Experimental Section

### 2.1. Materials

**Figure 1:**
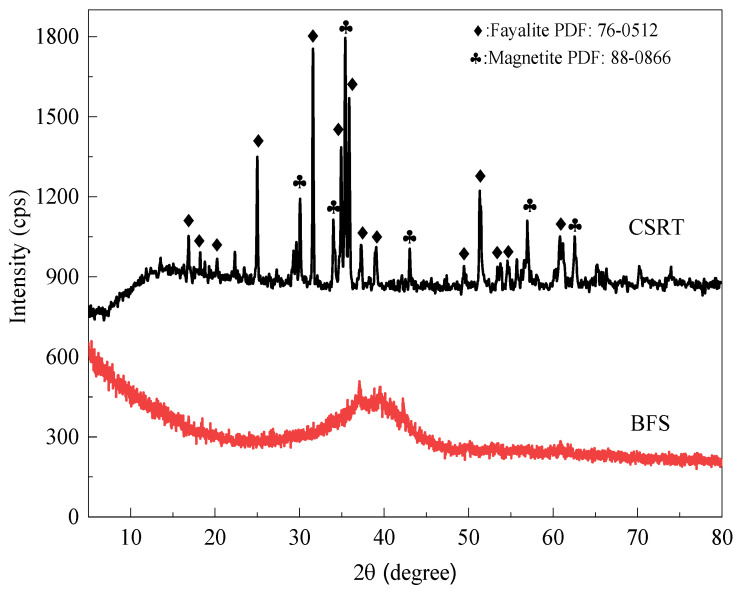
XRD patterns of the CT and BFS.

CT was obtained from the copper slag reprocessing tailings of Zijin Shan, Longyan, Fujian province, China, BFS from Tangshan iron and Steel smelter in Tangshan, Hebei province, China. Size distribution of CT and BFS was measured by a laser diffraction analyzer (LS-CWM, Omec, Zhuhai, China), in which the d_50_ and d_85_ were 16.81 and 36.71 µm for CT, and 11.18 and 26.30 µm for BFS, respectively. Table 1 shows the chemical composition of CT and BFS analyzed by an X-ray fluorescence instrument (XRF, Axios advanced, PANalytical B.V., Almelo, The Netherlands). The CT mainly contains SiO_2_ (28.19%) and Fe_2_O_3_ (57.83%), while CaO (38.55%), SiO_2_ (30.57%) and Al_2_O_3_ (15.09%) are the main components of the BFS. Figure 1 shows the main phase of the CT and BFS characterized by X-ray diffraction (XRD, D8, Brucker, Karlsruhe, Germany). The CT mainly contains magnetite (Fe_3_O_4_, PDF: 01-075-1609) and fayalite (Fe_2_O_4_Si, PDF: 98-001-0116), while BFS shows an amorphous peak at 2θ of 35–45°. Sodium silicate (ACS reagent grade), employed as an alkali activator, was bought from Aladdin Chemical Reagent (Shanghai, China) in the geopolymeric process. Deionized water was used in the whole process.

### 2.2. Methods

To synthesize the geopolymer, sodium silicate solution (6.17 M) was used as an alkaline activator and mixed with raw materials for 10 min. The raw materials included 30 g of CT and BFS, in which BFS proportions varied from 30% to 70%. Subsequently, the mixture was poured into cubic steel molds (30 mm × 30 mm× 30 mm), vibrating to liberate air bubbles on a vibration table for 3 min. Next, the molds were sealed with plastic bags and cured at 60 °C for 6 h and left at room temperature for 7 days to complete the hydration process and obtain initial geopolymers. After that, the initial geopolymers were exposed to five typical environments for 28 days: (1) in the air; (2) in deionized water; (3) in seawater; (4) in freeze–thaw seawater cycles, i.e., geopolymers were alternately exposed to seawater at room temperature for 12 h and 12 h in a freezer (−18 °C), and (5) concrete carbonation box (Temperature ±20 °C, humidity ±70%, CO_2_ concentration ±20%). The compressive strength of the geopolymers was analyzed by a compression tester (YAW-300, Jinan, China).

### 2.3. Characterizations

The compressive strength of the geopolymers was analyzed by a YAW-300 compression and flexure machine from Jinan Tianchen manufacture (Jinan, China). The morphology and microstructure of the geopolymers were characterized using a scanning electron microscope (SEM, Quanta 250, FEI, Hillsboro, OR, USA) and XRD diffractometer. The geopolymer was ground to less than 75 μm to prepare specimens for XRD measurements, in which Cu-Ka1 radiation and a scanning rate of 0.1°/s from 5° to 90° of 2θ were used. The geopolymers were studied using a Fourier-transform infrared (FTIR, Nicolet, Thermo Fisher Scientific, Waltham, MA, USA) spectroscopy, of which transmittance spectra were obtained over a wavenumber of 400–4000 cm^−1^; the ^29^Si nuclear magnetic resonance (NMR) spectra of the geopolymers were obtained via NMR spectroscopy (Bruker AVANCE III), whereby powdered samples were packed into 7 mm ZrO_2_ rotors. The spectra were acquired at spinning speeds of 12 kHz with peak positions referenced to an external standard of tetra-methylsilane (TMS). The lack of spectral resolution for silicon in geopolymers was overcome by implementing Gaussian peak deconvolution to separate and quantify Qn (mAl) species (0 ≤ m ≤ n ≤ 4, m, n = integer).

## 3. Results and Discussion

### 3.1. Compressive Strength

**Figure 2:**
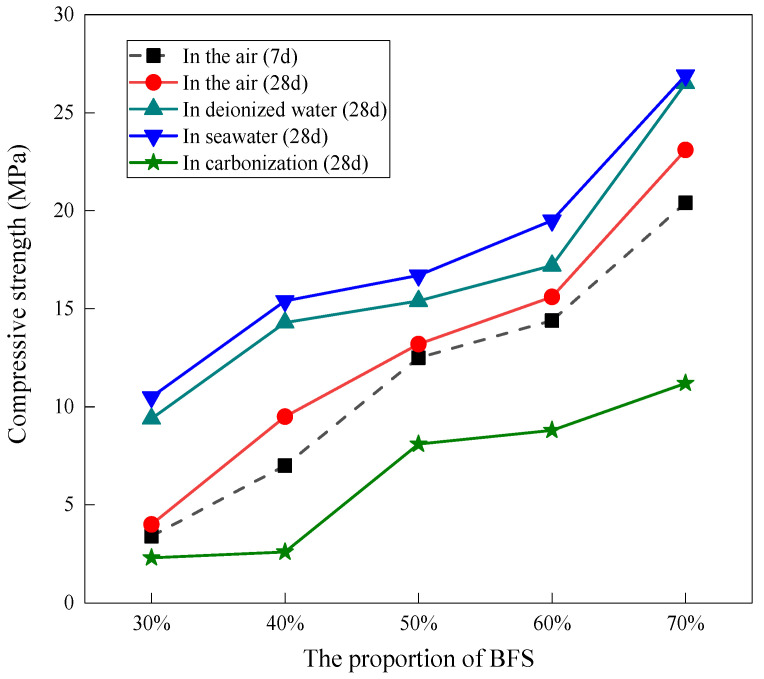
Compressive strength of the geopolymers prepared with varying proportions of BFS in different exposure conditions.

Figure 2 shows the compressive strength of geopolymers prepared with varying proportions of BFS in different exposure conditions. The initial geopolymers, cured for 7 days in the air, had compressive strengths of 3.4, 7, 12.5, 14.4 and 20.4 MPa as the proportion of BFS varied from 30 to 70%, respectively. This is attributed to the high pozzolanic reactivity of the aluminum and silicon components in the BFS, thus offering more precursors and generating more CaO-Al_2_O_3_-SiO_2_-H_2_O (C-A-S-H) and (Na, K)_2_O-Al_2_O_3_-SiO_2_-H_2_O (N-A-S-H) in the geopolymer with higher BFS content. Compared to the initial geopolymers, the compressive strength of the geopolymers, irrespective of the content of BFS, slightly increased after 28 days of exposure to air. This indicates that the geopolymeric reaction hydrated rapidly in the initial 7 days, which is consistent with a high early-age geopolymer compressive strength, as observed in other studies [22]. When geopolymers were exposed to deionized water for 28 days, the compressive strength increased significantly to 9.4, 14.3, 15.4, 17.2 and 26.5 MPa with 30, 40, 50, 60 and 70% BFS addition, respectively. Compared to exposure to deionized water, the compressive strength of the geopolymers exposed to seawater for 28 days further increased to 10.5, 15.4, 16.7, 19.5 and 26.9 MPa with 30, 40, 50, 60 and 70% BFS addition, respectively. However, the geopolymers exposed to the carbonized environment for 28 days exhibited the lowest compressive strength regardless of the curing age and BFS content. It is worth noting that the geopolymer exposed to the freeze–thaw seawater cycle showed the most severe deterioration, which failed to be subjected to a compressive strength measurement. Several factors could be responsible for these results. Firstly, the geopolymers exposed to an environment with water (deionized water and seawater) favored stability and mitigated the shrinkage of the geopolymers, thereby reducing the generation of harmful cracks and improving the compressive strength [23]. Secondly, in the carbonized environment, the intrusion of acidic CO_2_ decreased the alkalinity of the pore solution in the geopolymers since it reacted with the alkaline activator, hindering the geopolymeric reaction from further proceeding , thus lowering the compressive strength. The chemical equations can be expressed as Equation (1). Finally, the variation in the physicochemical environment may be related to the nanostructural and physical property changes, which governed the compressive strength changes of the geopolymers.

(1) $$\mathrm{Ca}{\left(\mathrm{OH}\right)}_{2}+H_{2}O+{\mathrm{CO}}_{2}\to {\mathrm{CaCO}}_{3}+2H_{2}O$$

### 3.2. Microstructure Characterizations

#### 3.2.1. Phase Transformation

**Figure 3:**
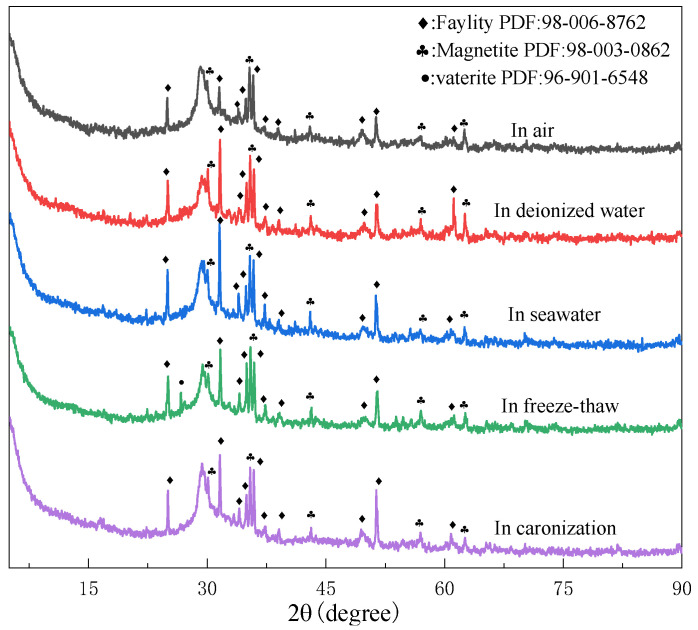
XRD patterns of geopolymers prepared with 60% BFS that underwent different exposures for 28 days.

The XRD patterns of the geopolymers prepared with 60% BFS subjected to different exposures for 28 days are shown in Figure 3. Compared to the XRD patterns of the raw materials, the crystal phase from the CT remained in the geopolymers, whereas the amorphous peak (2θ of 35–45°) from the BFS disappeared. Instead, a semi-crystal phase at 2θ of 29.3 was formed, which is the characteristic peak of the C-(A)-S-H gel [24,25]. Differently, a newly formed phase at 2θ of 27°, which is assigned to vaterite (Ca_4_C_4_ O_12_, PDF: 96-901-6548), was found in the geopolymer exposed in the freeze–thaw cycle. This may be due to the dissolved matter in the seawater precipitating and crystallizing when the water freezes in the geopolymer capillaries in the process of the freeze–thaw cycle [26], which might be related to the severe deterioration of the geopolymer exposed to the freeze–thaw cycle. In our previous study [27], significant peaks for the NaCl crystal phase exhibited in the XRD of the geopolymer exposed to artificial seawater. However, in the present study, few peaks in the NaCl crystal phase were found. This may be because the natural seawater is of a lower salt concentration compared to the artificial seawater. To shorten the experiment period, a high concentration of NaCl solution (5–10 fold of standard seawater) was used to accelerate the corrosion of the geopolymer matrix. From the XRD analysis, one can conclude that the natural seawater has little influence on the phase transformation of the geopolymer matrix, which indicates the merit of the geopolymer matrix compared to the OPC matrix. When the OPC matrix was exposed to the marine environment, the Ca-bearing phase would react with ions in the seawater to form Friedel’s salt (3CaO·Al_2_O_3_·CaCl_2_·10H_2_O) [27], Kuzel’s salt (3CaO·Al_2_O_3_·0.5CaCl_2_·0.5CaSO_4_·10H_2_O) [28], Ettringite (3CaO·Al_2_O_3_·3CaSO_4_·32H_2_O) [29], calcium oxychloride (CaCl_2_·3Ca(OH)_2_·12H_2_O) [30], and magnesium-silicate-hydrate (M-S-H) [31], and subsequently deteriorate the OPC concrete.

#### 3.2.2. FTIR Analysis

**Figure 4:**
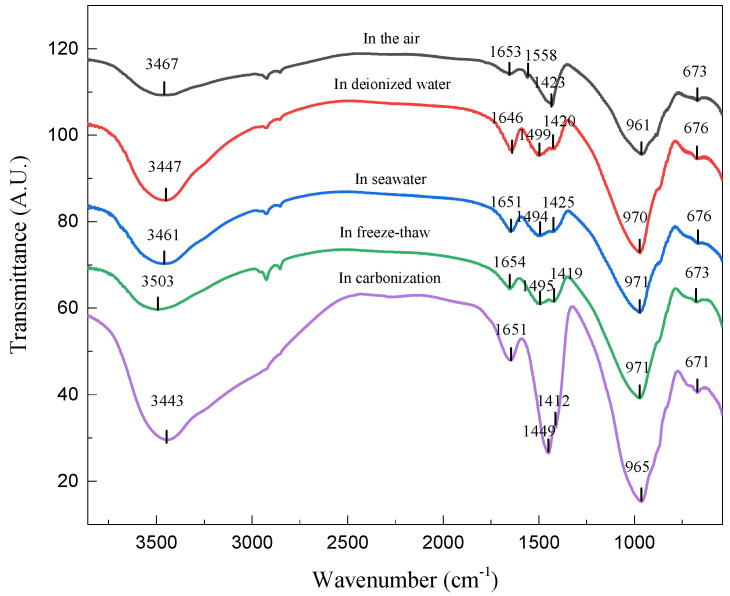
FTIR spectra of the geopolymers prepared with 60% BFS subjected to different exposures for 28 days.

Figure 4 shows the FTIR spectra of the geopolymers prepared with 60% BFS subjected to different exposures for 28 days. The absorption peaks at 3440 cm^−1^ and 1650 cm^−1^ are related to the stretching vibrations of the OH^−^ and H-OH bonds in the free water, respectively, corresponding to adsorbed H_2_O in geopolymers [32]. The absorption peak around 1420 cm^−1^ is assigned to asymmetric stretching of the O-C-O bonds in CO_3_^2−^ groups due to carbonation in the curing and exposure process of the geopolymers [33]. As can be seen, the absorption peak for the O-C-O bond is much more intense in geopolymer exposed to the carbonized environment than those in other environments. The peak at 673 cm^−1^ is attributed to the Si-O bonds in quartz, suggesting the quartz particles are insoluble in the geopolymeric reaction [34]. The band at about 970 cm^−1^ is the Si-O-T bonds (T represents the tetrahedral Al or Si) in the geopolymer gel [35]. The wavenumber transformation indicates that the varying exposure changed the C(N)-A-S-H gel evolution.

#### 3.2.3. NMR Analysis

**Figure 5:**
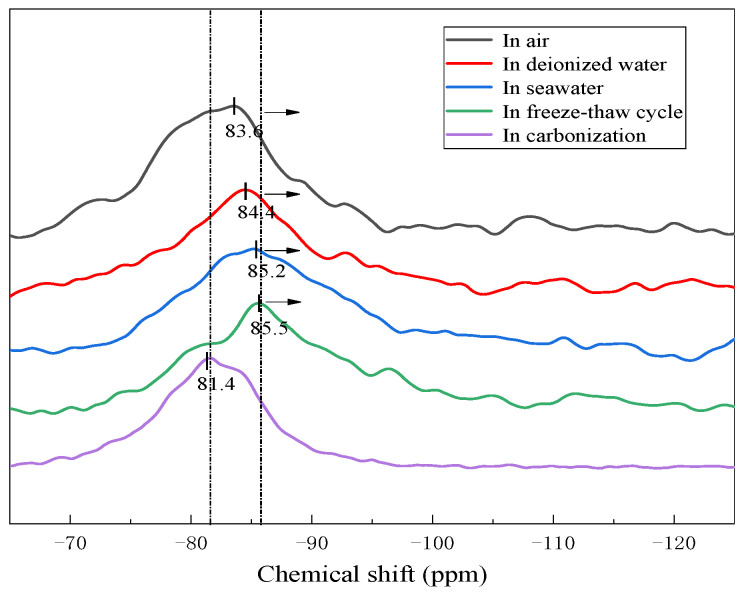
^29^Si NMR spectra of the geopolymers prepared with 60% BFS subjected to different exposures.

For further characterization of the nanostructure change in the geopolymers with different exposures, NMR spectroscopy was used to characterize the short-range ordering and molecular structure of the geopolymer [6,36]. It employs Gaussian peak deconvolution to overcome the lack of spectra resolution and to separate and quantify Q^n^(mAl) species (0≤ m ≤ n ≤ 4, m, n = integer). The resonances at −74, −79, −87.8 and −96.4 ppm are assigned to Q^0^, Q^1^, Q^2^ and Q^3^, presented in the binder C-(A)-S-H, respectively [37]. In N-A-S-H gel, the Q^4^(4Al), Q^4^(3Al), Q^4^(2Al), Q^4^(1Al) and Q^4^(0Al) resonate at around −84, −89, −93, −99 and −108 ppm, respectively [25,38]. The resonance at −104 ppm represents Q^3^ (R) when the H in OH is substituted by an alkali metal ion (Na^+^ or K^+^) in Q^3^ [18]. Figure 5 gives the ^29^Si NMR spectra of the geopolymers prepared with 60% BFS that underwent different exposures. As can be seen, the ^29^Si NMR spectra of the geopolymers exhibited a broad resonance ranging from δ_iso_ −75 to −95 ppm. However, the line shapes of distribution are different. This suggests that the varying structures were exhibited in geopolymers with different exposures.

**Figure 6:**
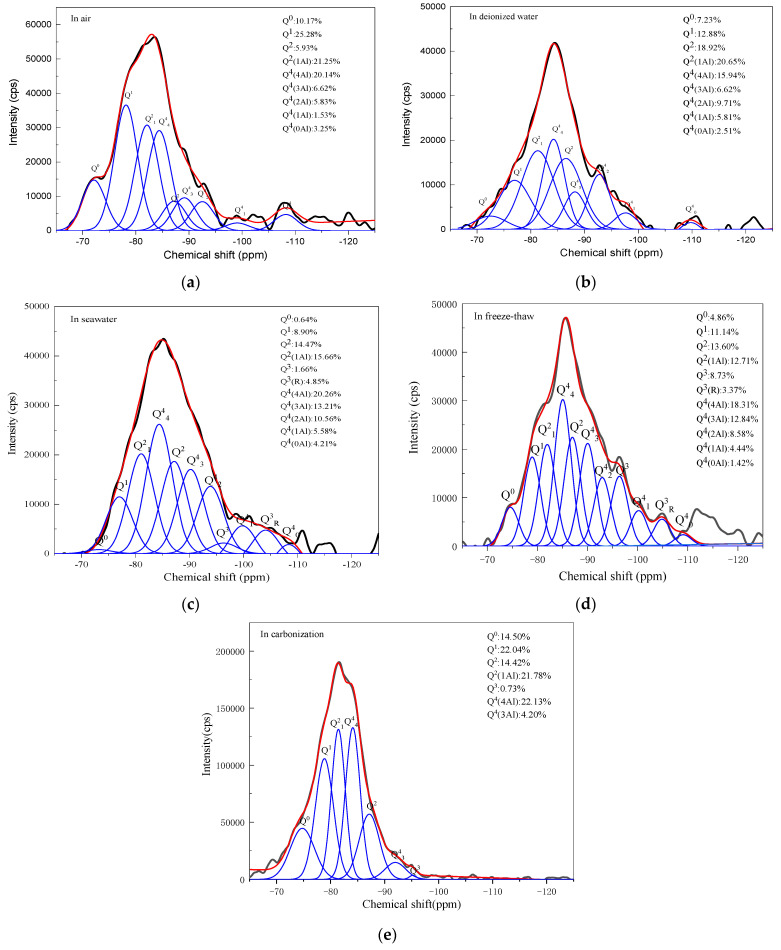
The deconvolution of ^29^Si NMR spectra of geopolymers prepared with 60% BFS subjected to different exposure.

Figure 6 gives the deconvolution of ^29^Si NMR spectra of geopolymers prepared with 60% BFS subjected to different exposures. For the geopolymers exposed to air, deionized water and seawater, the Si sites in the silicate monomers (Q^0^) and silicate dimmer (Q^1^) are 35.45%, 20.11% and 9.54%, respectively; the Si sites in the C-(A)-S-H (Q^2^ and Q^3^) and N-A-S-H gel (Q^4^) are 64.55%, 79.89% and 90.46%, respectively. The sites in the monomer and dimmer, to some extent, represent the unreacted silica source in the raw materials. Therefore, exposure to the water environment promoted the reaction extent and generated more cementitious gel, thus increasing the compressive strength of the geopolymers exposed to the deionized water and seawater. Compared to air exposure, the geopolymer exposed to the freeze–thaw cycle also exhibited fewer Si sites in Q^0^ and Q^1^ (16.00%) and more Si sites in Q^2^, Q^3^ and Q^4^ (84.00%), which was expected to have higher compressive strength. However, the compressive strength exhibited the most severe deterioration. This indicates that the compressive strength deterioration had nothing to do with the change in the chemical properties of the geopolymer (this will be further discussed later). For the geopolymer exposed to the carbonization environment, less N-A-S-H gel was formed than with exposure to the air, deionized water, seawater and heat–cool cycle. On the other hand, Q^4^(2Al), Q^4^(1Al), and Q^4^(0Al) disappeared in the geopolymer exposed to the carbonation environment, whereas the proportion of Q^0^ and Q^1^ (36.54%) increased. This indicates that the carbonation environment weakened the geopolymer evolution and decreased the cross-linking degree of the Q^n^(mAl) species in the N-A-S-H gel. It is worth noting that the proportion of Q^4^(4Al) (22.13%) still retained a high level in the geopolymer exposed to the carbonation environment, which may be due to the earlier formation of Q^4^(4Al) than Q^4^(2Al), Q^4^(1Al) and Q^4^(0Al). It has been reported that aluminum has a higher initial dissolution rate than silicon, resulting in the formation of an Al-rich gel in the initial stage of geopolymeric reaction [39], followed by the formation of Si-rich gel as the stoichiometric release of silicon. However, the carbonation environment decreased the alkalinity of the pore solution in the geopolymers due to the CO_2_ reacting with the alkaline activator, hindering the geopolymeric reaction from proceeding and the formation of Si-rich gel (Q^4^(2Al), Q^4^(1Al), as well as Q^4^(0Al)) in the later stage. Therefore, the compressive strength decreased when the geopolymer was exposed to the carbonation environment, as demonstrated in the results shown in Figure 2.

#### 3.2.4. Morphological Analysis by SEM

**Figure 7:**
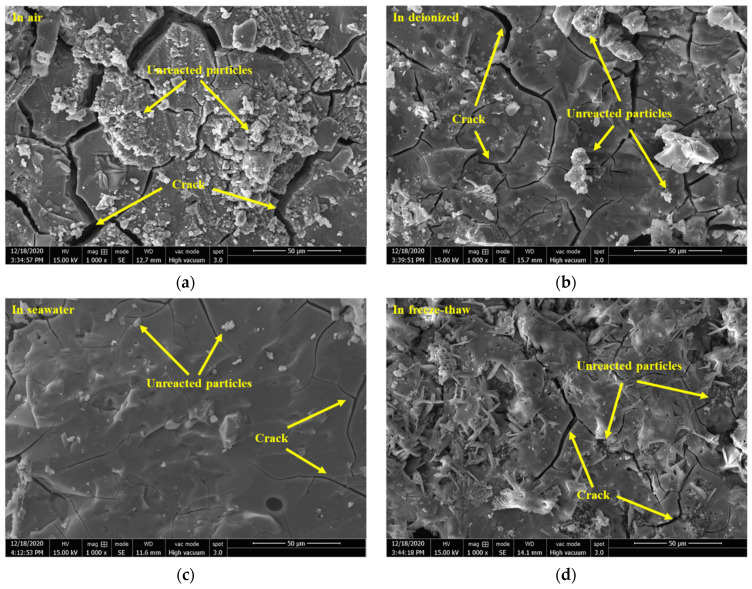

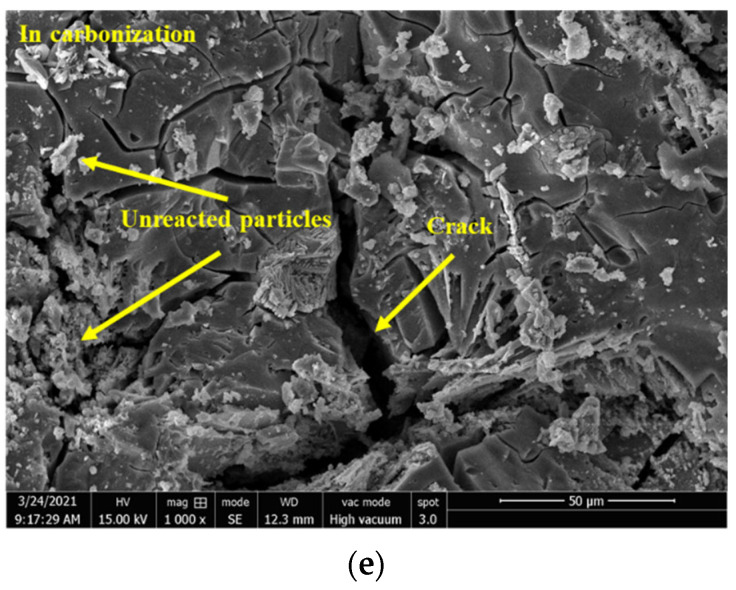
SEM images of the geopolymers prepared with 60% BFS subjected to different exposure.

Figure 7 gives the SEM images of the geopolymers prepared with 60% BFS subjected to different exposure. The geopolymers exposed to carbonization and air show numerous deep cracks and unreacted particles, in which the cracks are more severe in the geopolymer exposed to carbonization than exposed to air. Cracks and unreacted particles were decreased in the geopolymer exposed to deionized water. The geopolymer exposed to seawater exhibited the slightest cracks and particles and homogenous and compact structures. This is related to the self-shrinkage of the BFS-based geopolymers. Due to the migration and dissipation of water inside the test piece to the outside world and the reduction of escapable water in the structural system during the hydration reaction, shrinkage, cracking and incomplete reaction of the test occurred. The two water environments, ionized water and seawater, provided good conditions for the subsequent polymerization reaction of the specimens, resulting in a smoother and more uniform matrix, as well as a higher reaction level. These results were consistent with the results of the compressive strength, as shown in Figure 2. Additionally, when the geopolymer was exposed to the freeze–thaw cycles, some star-shaped substances were formed in the matrix. This may be related to the crystallization of the geopolymers exposed to the freeze–thaw cycles, as shown in Figure 3.

#### 3.2.5. Macromorphology Analysis of Geopolymer Exposed to Freeze–Thaw Cycle

**Figure 8:**
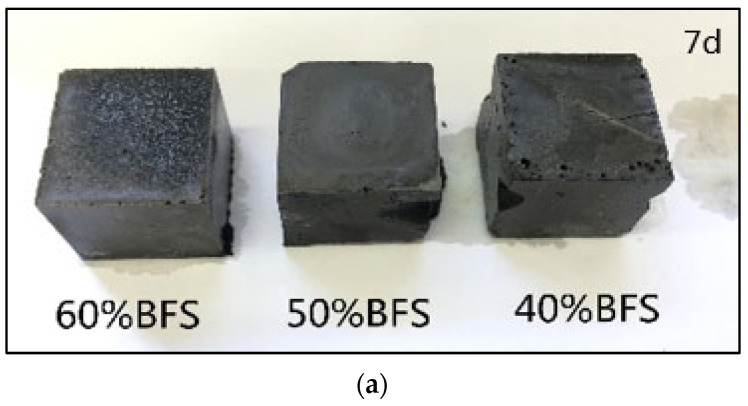

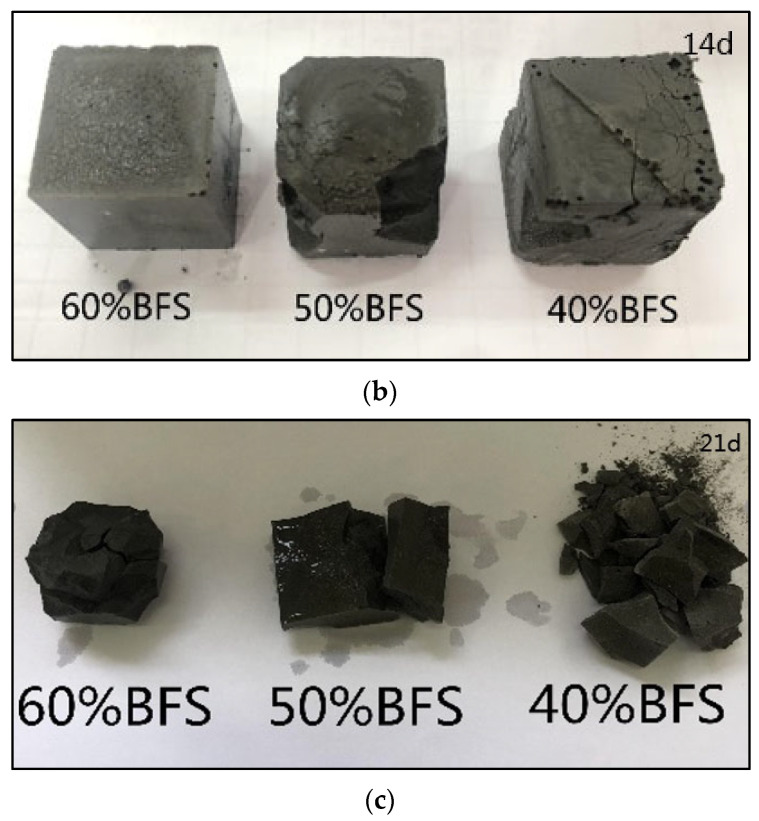
Macro changes in geopolymer prepared with different proportions of BFS overtime under freeze–thaw cycle.

The geopolymer exposed to the freeze–thaw cycle exhibited the most severe deterioration. Figure 8 gives the macro changes of the geopolymer prepared with different proportions of BFS over time under the freeze–thaw cycle. Compared with the freeze–thaw cycle of 7 days, the 14-day geopolymer started to show cracks and edge peeling. On the other hand, after 21 days of curing the geopolymer, all the blocks collapsed and the geopolymers showed an unconsolidated matrix. In the freeze–thaw cycle, water saturating, freezing, swelling and melting in the cracks take place reduplicative, thus accelerating the decomposition of the geopolymer matrix. Therefore, it failed to obtain the 28-days compressive strength value. Compared to the three samples with different BFS content, the anti-freeze–thaw effect is better when the BFS content is 60%. The addition of BFS increases the content of CaO and generates more C-(A)-S-H gels. The NMR analysis found that geopolymers exposed to the freeze–thaw cycle have fewer Si sites in Q^1^ and Q^2^ and more Si sites in Q^2^, Q^3^ and Q^4^ compared to that exposed to the air, which had a similar evolution trend to those exposed to air, deionized water and seawater. Geopolymers exposed to deionized water and seawater exhibited higher compressive strength than those exposed to the air. In contrast, the geopolymer exposed to the freeze–thaw cycle showed the most severe deterioration. This result indicates that the deterioration had nothing to do with the evolution of the chemical nanostructures but rather with the changes in physical property. From the XRD analysis, it was found that an additional crystal phase (vaterite) was formed in the geopolymer exposed to the freeze–thaw cycle, which may have resulted in the deterioration of the geopolymer due to the formation of cracks caused by the crystallization degree exceeding the ultimate tensile strength of the geopolymer [40].

## 4. Conclusions

This work studied the gel evolution of the CT-based geopolymer in the marine environment. It was found that deionized water and natural seawater exposure promoted the evolution degree of the geopolymers, in which the seawater exhibited a greater promotion effect. The increased gel formation improved the compressive strength of the geopolymer. The carbonization environment exposure weakened the gel evolution and decreased the cross-linking degree of the N-A-S-H gel nanostructure, resulting in decreased compressive strength. The geopolymer exposed to the freeze–thaw cycle exhibited the worst deterioration due to the expansion caused by crystallization in the geopolymer. On the other hand, geopolymer concrete shows excellent properties in marine-related environments. This provides the possibility for the practical application of geopolymer concrete in coastal breakwaters and other buildings, as well as the opportunity to transform solid waste from conventional consumption and disposal and low-value-added brick-making into a high-value utility. 

## Figures and Tables

**Table 1:** Chemical composition of the CT and BFS.

Components (%)	SiO_2_	Al_2_O_3_	Fe_2_O_3_	MgO	CaO	TiO_2_	Na_2_O	K_2_O
CT	28.19	4.24	57.83	1.101	1.906	0.303	0.525	0.81
BFS	30.57	15.09	0.33	1.305	38.55	1.60	0.50	0.37

## Data Availability

Not applicable.
